# Experimental study of the function of the excreted/secreted *Leishmania *LmSIR2 protein by heterologous expression in eukaryotic cell line

**DOI:** 10.1186/1475-9292-4-1

**Published:** 2005-01-24

**Authors:** Denis Sereno, Laurent Vanhille, Baptiste Vergnes, Adriano Monte-Allegre, Ali Ouaissi

**Affiliations:** 1 UR008 "Pathogénie des Trypanosomatidés", Centre IRD de Montpellier, 911 avenue Agropolis, BP5045, 34032 Montpellier, France

## Abstract

**Background:**

In yeast and *Caenorhabditis elegans*, Silent Information Regulator (SIR2) proteins have been shown to be involved in ageing regulation. In *Leishmania*, the LmSIR2rp was originally isolated from the excreted/secreted material of the *Leishmania *parasites. Among the function(s) of this protein in *Leishmania *biology, we have documented its implication in parasite survival, and in particular in *Leishmania *amastigotes. In this paper we question the role of the excreted/secreted form of the protein. In particular we wonder if the *Leishmania *Sir2 homologue is involved in some aspect of its biological function(s), in various components and pathways, which could promote the host cell survival. To test this hypothesis we have mimicked an intracellular release of the protein through constitutive expression in mouse L929 fibrosarcoma cells.

**Results:**

Our results demonstrate that the LmSIR2 protein was properly expressed by fibroblasts and that LmSIR2 is localized both in the cytoplasm and the nucleus of all the transformed cell clones. Unexpectedly, we found that cells expressing LmSIR2 presents reduced saturation cell density ranging from 40% to 60% and expressed an acidic (pH6.0) β-galactosidase activity, which is known to be a senescence biomarker. As a consequence, we observed that LmSIR2 positive fibroblasts were more permissive towards *Leihmania *infection.

**Conclusions:**

LmSIR2 is able to substantially interfere with the host cell physiology. Thus, it is tempting to speculate that these modifications could help *Leishmania *to survive for a long period in a cell with reduced capacity to multiply or respond to immunologic stimuli. The potential implications of our finding during the *in vivo *infection process are discussed.

## Background

*Leishmania *is an intracellular pathogen that causes Leishmaniasis, which remain an important medical problem in several countries. Protozoan parasites of the genus *Leishmania *result in a spectrum of human disease that range from self-healing cutaneous ulcers to potentially fatal visceral infection, depending primarily upon the species of parasite involved [1,2]. *Leishmania *live as either extracellular flagellated promastigotes in the digestive tracts of their sand fly vectors or as nonflagellated amastigotes within macrophages where they survive and replicate within phagolysosome. *Leishmania *are known to export large range of proteins and glycoconjugates including lipophosphoglycan [3].

In a previous paper we have characterized a *Leishmania major *gene encoding a protein carrying extensive homology to SIR2 of yeast that we consequently termed LmSIR2rp [4]. It belongs to a large family of closely related NAD-dependent deacetylase named Hst proteins (Homologous of Sir two) or sirTuins, present in both prokaryotic and eukaryotic species [5]. Post-translational modifications of histones, like acetylation by histone acetylase (HAT) and deacetylation by histone deacetylase (HDAC), within the context of chromatin has been shown to regulate gene expression [6,7]. To date, three classes of HDACs are characterized in eukaryotes, the class I and II HDACs, and the class III defined by the sir2 family. In eukaryotic species some members of the Sir2 family are much more closely related to the core domain of the yHst2p protein than to the core domain of ySir2p itself [8]. These are: the SIR2 family members from *Schizosaccharomyces pombe*, *C albicans*, *Leishmania*, chicken, human (hSirT2p, hSirT3p) and the closely related mouse protein (MmSirT2p and MmSirT3p) [8]. This group has been designated Hst2-like, as it forms an independent branch within the Sir2 family tree. Five members of this group (LmSIR2rp), like it yeast (yHst2) human (SIR2L) and mouse (MmSIR2L2 and MmSIR2L3), are cytoplasmic [4,9,8,11]. The implication of SIR2 proteins bearing nuclear localization, in aging process has been extensively studied. In yeast, the deletion of SIR2 shortens lifespan and over expression extends lifespan [12]. This phenotype of life extension has been also observed in *Caenorhabditis elegans *carrying extra copies of the SIR2 orthologue, Sir-2.1 [review in [13]]. In mammalian cell, SIRT1 promote cell survival [14]. SIRT1 is able to deacetylate FOXO3 and/or FOXO4, thus attenuating FOXO-induced apoptosis and potentiating FOXO-induced cell-cycle arrest. Thus SIRT1 might increase longevity by shifting FOXO dependent responses away from cell death and toward cell survival [15]. Also, the unusual NAD-requirement for the Sir2 deacetylase may link metabolic rate to silencing and life span [16].

We have shown that the cytosolic LmSIR2rp protein, when over expressed in *Leishmania *amastigotes, was able to promote parasite survival in *in vitro *culture systems [9]. Further, we also observed that LmSIR2rp can be detected in the excreted/secreted material of *Leishmania *parasites when using radiolabelled immunoprecipitation technique, suggesting therefore that fraction of LmSIR2 is actively excreted by parasite (personal observations). We then initially surmised that this protein could also be involved in some aspect of host cell-parasite interplay, particularly in pathways that could contribute to cell survival. To test this hypothesis, the *leishmania *LmSIR2 gene was cloned in a mammalian shuttle vector and the expression of the protein was performed in fibroblasts. Unexpectedly, expression of the *Leishmania *Sir2 protein leads to a cell growth arrest phenotypes, which correlates, with the expression by the cells of an acidic (pH 6.0) β-galactosidase activity, known to be a senescence biomarker. Furthermore, fibroblasts expressing LmSIR2 were more permissive towards *Leishmania *infection than control ones. Altogether our results may suggest that the excreted forms of LmSIR2 could participate in the perturbation observed during chronic disease.

## Materials and Methods

### Molecular construct and transfection procedure

*LmSIR2 *was amplified using sense GAA TTC GAT ATG ACA GGG TCT CCG and antisense CTC GAG CAG TCA CCA TGT TGG CAG primers. The 1119 bp PCR amplified genomic fragment subcloned into pCR2.1 was digested with *Eco*RI and inserted into the *Eco*RI digested and dephosphorylated pcDNA3.1 shuttle vector (Amersham). Restriction analysis of the recombinant plasmids using *Eco*RI and *Xho*I allowed the identification of recombinant vector carrying the *LmSIR2 *gene in sense orientation. Large-scale preparations of pcDNA-*LmSIR2 *and pcDNA empty vectors were performed using the QIAGEN plasmid maxi kit. Transfected cells were selected in RPMI 1640 medium containing 10% FBS and 400 μg/ml G418. Each G418-resistant clone, isolated by limiting dilution, were propagated and screened by immunofluorescence. Four clones were isolated and named Cl2, Cl9, Cl10 and Cl11.

### Production of monoclonal antibodies (mAb) against the fusion protein

LmSIR2 recombinant protein containing 6 X histidine residues at its N-terminal (hisLmSIR2) was previously obtained after having subcloned the encoding gene in the pQE-expression vector [17]. Mice of BALB/c strain were injected intraperitoneally with 50 mg of hisLmSIR2 in saline solution emulsified with 0.1 ml of Freud's Complete Adjuvant. After 2 weeks, a booster injection of the same preparation was given. The antibody response of the animals was tested 1 month later. The same dose of antigen was injected intravenously 3 days before the hybridization experiment.

The fusion procedure and the screening of mAb were carried out as previously described [18]. The class of mAb was determined by agarose double diffusion of supernatants against antisera specific for immunoglobulin subclasses obtained from cliniscience (Montrouge, France). Several hybridoma clones producing mAb reacting in an ELISA test with LmSIR2 protein were obtained; only one mAb of the IgG1 isotype (IIIG4) reacted in Western blot against the fusion as well as the native LmSIR2 protein.

### Growth kinetic and ^3^H-thymidine incorporation

L929 cells were inoculated at 400 cells/cm^2^, after various period of incubation they were collected by using Trypsin-EDTA (0.025%). The mean number of viable cells was determined at 100-× magnification after staining with trypan blue. In order to evaluate the rate of ^3^H-thymidine incorporation, cells were seeded in 96 wells plates at 1000 cells/cm2, after various periods of incubation 1 μCi of ^3^H-thymidine was added to each well. Cells were further incubated for 4 hours before reading the level of incorporated radioactivity.

### Giemsa staining and Immunofluorescence analysis

L929 cells were seeded in 16-well Labteck chambers for 48 hrs. Cells were then washed with PBS and fixed for 30 minutes with 4% paraformaldehyde at 4°C. After two washes with PBS, cells were permeabilized with 0.01 M PBS containing 0.5% TritonX-100 and 2% BSA for 30 min at 4°C. Slides were then washed and incubated for 1 hour with mAb IIIG4 in 0.01 M PBS containing 2% BSA. After two washes, slides were incubated for 45 min with fluoresceine-conjugated rabbit anti-mouse IgG (Diagnostic Pasteur, Marnes la Coquette, France) diluted 1:100 in PBS containing Evan's blue (final dilution in PBS: 1: 10.000) and mounted in glycerol PBS (1:1).

Giemsa staining of the cell culture was performed as follows: cells were inoculated at 400 cells/cm^2 ^in 25 cm^2 ^flasks, after 7 days of growth they were fixed with methanol and stained with Giemsa and examined observed on inverted microscope (40× magnification) after adding a solution of PBS 0,01 M-Glycerol (1/1).

### RT-PCR analysis

Total RNA was isolated from wild type and transformed cells using the Rneasy Mini Kit following the manufacturer's instructions. One microgram of RNA was reverse transcribed to cDNA with an oligonucleotide [poly (dT)_12–18_] using the Superscript II RNase H-reverse transcriptase. PCR amplifications were performed using 1–2 μl of reverse-transcription of each product and 20 pmol of each primer, using Taq DNA polymerase. Each PCR cycle consisted of a denaturation step (94°C, 1 min), an annealing step (55°C, 1 min) and an elongation step (72°C 1 min). For the last cycle, the elongation step was extended to 10 min at 72°C. Reactions were carried out for 25–35 cycles in a thermocycler (PTC-100™, MJ Research, Inc.). PCR products were analyzed on 1.5% agarose gel and visualized with ethidium bromide. As an internal control, a housekeeping gene, the glyceraldehyde-3-phosphate dehydrogenase (GAPDH) transcript, was amplified. *LmSIR2 *primers were: Sense GAA TTC GAT ATG ACA GGG TCT CCG and antisense CTC GAG CAG TCA CCA TGT TGG CAG. GAPDH primers were: Sense GTC TTC ACC ACC ATG GAG and antisense CCA AAG TTG TCA TGG ATG ACC.

### β-galactosidase pH6 analysis

Acidic (pH 6.0) β-galactosidase was assayed according to Dimri et al. [19]. Briefly, stationary-phase cells were washed three times in phosphate buffered saline (0.1 M PBS, pH 7.4), fixed in 2% formaldehyde/0,2% glutaraldehyde for 5 min at room temperature, washed three times with PBS, then incubated at 37°C without CO_2 _for 12 h with freshly prepared β-Gal staining solution pH 6.0, containing the 5-bromo-4-chloro-3-indoyl-b-D-galactosidase (X-Gal) chromogenic substrate which produces blue 5-bromo-4-chloro-3-indole upon hydrolysis. Cell preparation was stored at 4°C in 70% glycerol upon analysis.

## Results and Discussion

### Expression of LmSIR2 by L929 fibroblasts

**Figure 1:**
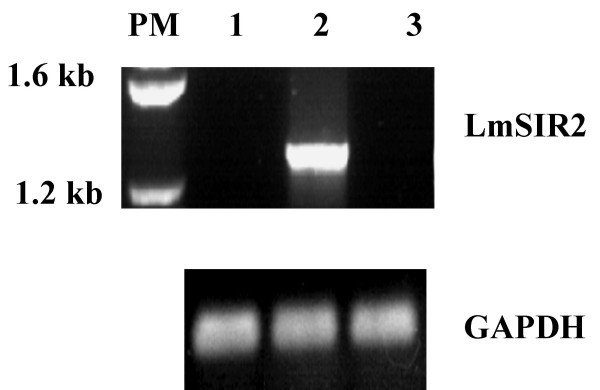
**Expression of LmSIR2 gene in L-929 cells. **RT-PCR analysis of total RNA from various L-929 clones was carried out using LmSIR2 or GAPDH primers. The lanes correspond to the following samples: standard size marker (PM); WT L929 cells (1); L929 clone 2 (Cl2) (2); L929 carrying an empty pcDNA plasmid (3).

**Figure 2:**
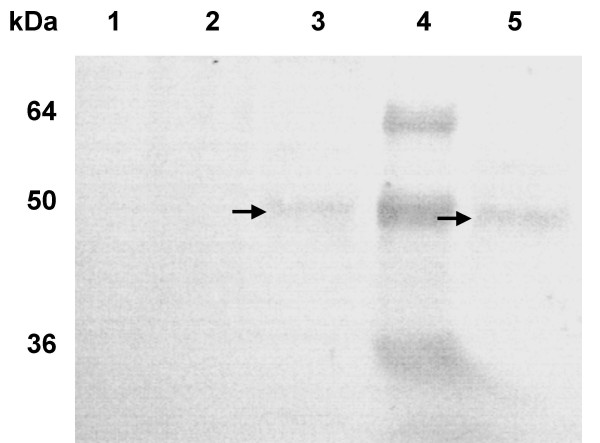
**Detection of the LmSIR2 protein in L929 cell extracts. **The lanes correspond to the following samples: WT L929 cells (1); L929 carrying an empty pcDNA plasmid (2), L929 Cl2 (3); Molecular weight markers (4); L-929 Clone 9 (5).

The Level of the LmSIR2 gene transcription was evaluated by RT-PCR analysis. As shown in Figure 1, an amplification product of around 1.2 Kb was observed only in recombinant L929 cells transformed with the pcDNA carrying LmSIR2 gene (Fig 1 lane 2), No amplification could be detected in Wild-type fibroblasts or in fibroblasts carrying the empty pcDNA vector (Lane 1 and 3 respectively). Accordingly, Western blot analysis of cellular extracts derived from the clones 2 and 11 reacted with with the mAb IIIG4 showed a polypeptide of 50 kDa (Fig 2 lanes 3 and 5), which is slightly higher than the molecular size of the pcDNA encoded protein (≈ 43 kDa). However, it is noteworthy that this molecular size is similar to that of the parasite native protein shown in previous studies [9]. It is therefore likely that the transfected gene product is submitted to posttranslational modifications that occur during its processing. No cross reactivity was observed on cell extract derived from Wild-type cell or fibroblast carrying the empty vector (Fig 2 Lane 1 and 2). Thus, these results confirm that mouse L929 fibroblast cell line properly expressed the *LmSIR2 *gene.

**Figure 3:**
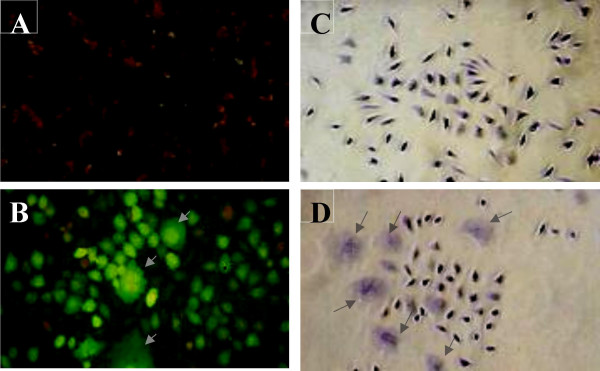
**Indirect immunofluorescence analysis and morphology of transgenic cells**. Localisation of LmSIR2 using immunofluorescence analysis (A) L929 cells carrying an empty pcDNA vector (B) L929 (Cl2) expressing LmSIR2 protein. Morphology of cells in culture: (C) L929 cells carrying an empty pcDNA vector and (D) L929 Cl2. Note the reduced cell density and the presence of cells bearing abnormal morphology (arrow).

The *LmSIR2 *gene was originally isolated via immunoscreening of a cDNA library, using polyclonal antibodies raised against excreted factors which could bind to glutathione [4]. The presence of such protein in the *leishmania*'s excreted material as previously demonstrated raised the question of its function during the infection process. Thus, heterologous expression in eukaryotic cells could represent a useful approach to give some insight into the biological activity of secreted parasite material. Immunolocalization of LmSIR2 shows that cells expressing LmSIR2 (Figure 3B) react with the monoclonal antibody as compared to cells carrying the empty pcDNA vector (Figure 3A). Interestingly, high number of cells positive for LmSIR2 bears abnormal morphology i.e. giant multinucleated cells (Figure 3B). In these cells, the protein is localized both in the cytosol and the nucleus. Functional studies of several mammalian SIR2 related proteins have been carried out including those who are mainly localized in the cytosol. Nevertheless, similar cellular localization pattern has never been reported nor such modification of cell physiology. Our observations suggest that LmSIR2 possesses unrelated specificity that can strongly affect the biological properties of the host cell. Interestingly, LmSIR2 protein presents a NES (the nuclear export signal or NES)-like motif LX_3_L_X_LX_3_L at its carboxy-terminal region, where L represents Leucine and X any amino acid [20]. The intracellular localization of key regulatory proteins tagged with a short Leucine-rich motif (nuclear export signal) is controlled by CRM1/exportin1, which is involved in the export of these proteins from the nucleus [reviewed in [21]]. However, the ability of the *Leishmania *SIR2 to shuttle between the cytoplasm and nucleus of the host cell through the NES putative sequence, await further investigations.

When L929 cell culture, of either clone 1, clone 2, clone 9 or clone 10, were stained with Giemsa, a high rate of cells with unusual shape and size associated with the presence of multiple nuclei was revealed (Figure 3 D) as compared to cells carrying the empty pcDNA vector (Figure 3C). The rate of multinucleated cells was determined by counting the number of cells bearing more than 2 nucleus per 10 champs at 10-× magnification. In fact all the clone cultures present a 10 to 15-fold increase in giant multinucleated cells as compared to cultures of L-929 fibroblast transformed with the empty vector (Figure 3 C). Moreover, majority of the cells if not all, present an enlarged and flattened morphology (Figure 3 D).

### Growth kinetic parameters of L929 cell expressing LmSIR2

**Figure 4:**
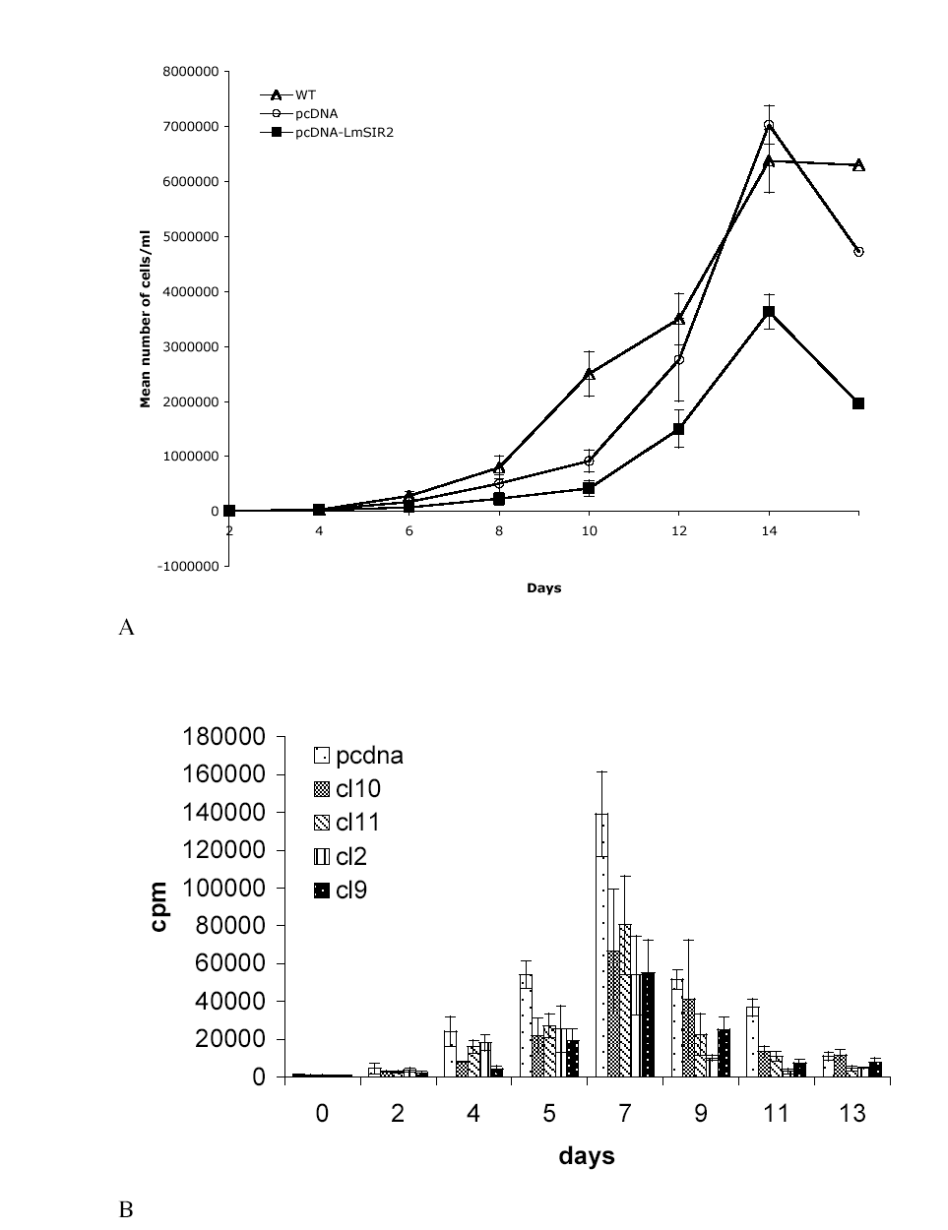
**Growth kinetics analysis. **Cell proliferation was determined using two methods: cell counting (A), the results are given as a mean value of a triplicate experiment ; and ^3^H-thymidine incorporation (B), the results are given as mean value of a sextuplate experiment

In order to investigate the potential functions of LmSIR2 protein, when delivered intracellularly in L929 fibroblasts, we have determined the growth kinetic parameters of cells via two different methodologies. Using a counting method, we observed a high reduction in the saturation density of cells expressing LmSIR2 (Figure 4A). The reduction observed in this experiment was as high as 40% as compared to cells carrying the empty pcDNA vector. These preliminary observations were further confirmed using ^3^H thymidine. Indeed, as shown in figure (4B), expression of LmSIR2 strongly reduced the thymidine incorporation over the time course of the experiment. The most dramatic reduction of 3H-thymidine incorporation was observed a day 7 which correspond to the maximal cell density observed for both Wild type control and the four clones studied (reduction in ^3^H-thymidine incorporation of about 61% for clone 2, 60% for clone 9, 52% for clone 10 and 42% for clone 11).

### Expression of senescence biomarker

**Figure 5:**
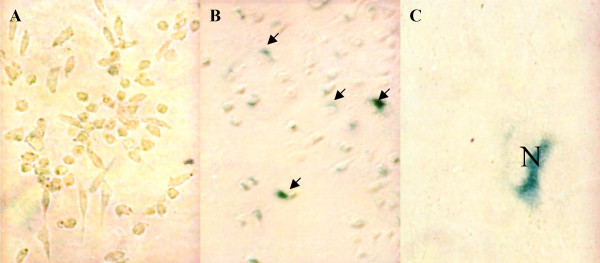
**Expression of a senescence biomarker. **(pH6.0) β-galactosidase activity detected in L929 cells expressing LmSIR2 (Cl2) (B and C) but not in L929 carrying an empty pcDNA plasmid (A). Cells were seeded at 40 cells/mm^2 ^in 25 cm^2 ^flasks and after 13 days of growth, the presence of pH6-galactosidase activity (Arrow) was determined as described in the Materials and Methods section. N nucleus, note the perinuclear galactosidase activity.

Parameters of senescence include increased cell size, reduced saturation density as well as altered expression of some gene products that have been proposed to serve as biomarkers of aging. Reduction in the final cell yield or saturation density is known to occur in human diploid fibroblast senescence and is considered as a criterion of the onset of senescence [22]. With respect to biomarkers, pH 6 β-galactosidase is particularly useful, since this activity has been found to distinguish senescent from quiescent cells both *in vivo *and *in vitro *[19]. We found that L929 cells expressing LmSIR2 exhibit reduced saturation densities (40% to 60%) and altered cell morphology. We have thus evaluated the occurrence of the pH 6 β-glactosidase activity. As shown on Figure 5 (B and C), a strong pH-6 β-glactosidase was detected in L929 cells expressing the *LmSIR2 *gene but was not detected in wild-type cells or cells transformed with the empty pcDNA vector (Figure 5A).

### Permissivity of fibroblast expressing LmSIR2 towards *Leishmania amazoensis*

**Figure 6:**
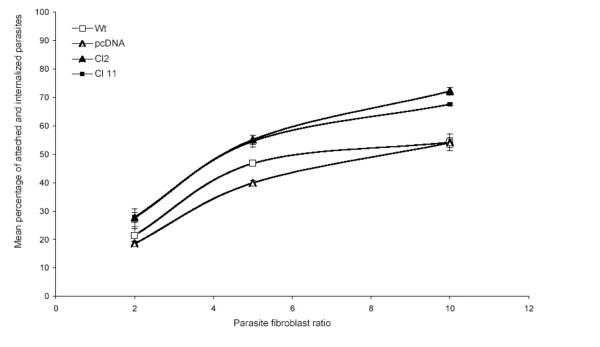
**Permissivity of L929 cells toward *Leishmania amazonensis *infection. **L929 cells were seeded in 25 cm2 flasks and treated with mitomycin. They were then infected, at different parasite/fibroblast ratio, with freshly differentiated stationary phase *L. amazonensis *promastigotes. After 24 hours of contact cells were washed and the percentage of fibroblats bearing attached or internalized parasites was determined. Results are expressed as a mean value of a triplicate experiment.

Fibroblasts at the lymph are used as safe target for long lasting residual parasite population [23]. However, only few reports deal with *in vitro *infection of fibroblast. In 1978, Chang [24] demonstrated that promastigotes of *L. amazonsensis *but not *L. donovani *were able to infect human skin fibroblasts. However, once differentiated into amastigotes the parasites were unable to multiply inside the cell. Dedet and co-workers further confirmed these observations [25]. Thus, we decided to test the capacity of *L. amazonensis *to infect L929 cell lines. When *L. amazonensis *promastigotes were incubated with Wild-type fibroblasts the proportion of infected cells reached a peak of about 50% after 24 hours of contact, after what the proportion of infected fibroblasts decreased quickly. On day 2, only few parasites could be seen and after 3 days of incubation less that 1% of fibroblasts remain infected (data not shown). As shown in Figure 6, fibroblasts expressing LmSIR2 were more permissive toward *Leishmania *infection since the mean percentage of infected fibroblast reach 70% after 24 hours of contact. However, once inside fibroblasts no differences in the capacity of parasites to survive and/or multiply was observed and the intracellular amastigote population was eliminated after three days of contact (data not shown).

## Conclusions

Our study demonstrates that LmSIR2 is able to down regulate the proliferation of fibroblasts and to induce a senescence-like state in L929 fibroblast cell line. The most interesting finding is that a parasitic protein is able to modify the physiology of the host cell making them more permissive toward *Leishmania *infection. These results suggest that LmSIR2 has dual function according to the cell type in which it acts: in *Leishmania *parasites, LmSIR2 is able to promote cell survival while in the host fibroblast it induces a senescence phenotype. This effect could be beneficial for *Leishmania *since senescent cells are no longer capable of dividing yet remaining metabolically active. They could thus represent safe target for parasite. It is documented that senescent fibroblasts present an inherent higher capacity to resist to apoptotic death [26]. In this view, it will be of interest to determine if fibroblasts that express LmSIR2 were more resistant to apoptotic stimuli. It is known that infected macrophages are usually more resistant to apoptosis, however it is not know if such mechanism operate in infected fibroblast. Thus, the implication of LmSIR2 in a general mechanism leading to apoptosis resistance of both macrophages and fibroblasts await further investigations. The *in vivo *relevance of our observation is rather difficult to appreciate because; (1) the level of the protein produced in fibroblasts transformed with the pcDNA is certainly far higher than the level achievable *in vivo*, and (2) the presence of excreted parasite material inside the fibroblast cytosol has yet not been reported. However, an action of the excreted form of LmSIR2 on he host cell could no be ruled out. Two series of observations support this possibility: (1) it has been shown that the *Leishmania *EF1-a could be translocated from the parasitophorous vesicle into the macrophage cytosol [27]; (2) the ultrastructural examinations of skin biopsies obtained from patients with cutaneous leishmaniasis showed that intracellular amastigotes could be frequently identified in vacuoles and in the cytosol of the host cell [28].

In conclusion, although the effect of the protein, when delivered extracellularly, on the host cells have not being examined in the present study, our data support the notion that the excreted form of LmSIR2 is able to interfere with the host cell physiology, when delivered intracellularly. Unexpectedly the expression of the protein is associated with the appearance of a senescence biomarker. Relevance of such finding during the *in vivo *infection process awaits further investigations

## References

[B1] Badaro R, Jones TC, Carvalho EM, Sampaio D, Reed SG, Barral A, Teixeira R, Johnson WD (1986). New perspectives on a subclinical form of visceral leishmaniasis. J Infect Dis.

[B2] Berman JD (1986). Human leishmaniasis: clinical, diagnostic, and chemotherapeutic developments in the last 10 years. Clin Infect Dis.

[B3] Ilg T, Stierhof YD, Wiese M, McConville MJ, Overath P (1994). Characterization of phosphoglycan-containing secretory products of *Leishmania*. Parasitology.

[B4] Yahiaoui B, Taibi A, Ouaissi A (1996). A *Leishmania major *protein with extensive homology to silent information regulator 2 of *Saccharomyces cerevisiae*. Gene.

[B5] Frye RA (2000). Phylogenetic classification of prokaryotic and eukaryotic Sir2-like proteins. Biochem Biophys Res Commun.

[B6] Strahl BD, Allis CD (2000). The language of covalent histone modifications. Nature.

[B7] Jenuwein T, Allis CD (2001). Translating the histone code. Science.

[B8] Perrod S, Cockell MM, Laroche T, Renauld H, Ducrest AL, Bonnard C, Gasser SM (2001). A cytosolic NAD-dependent deacetylase, Hst2p, can modulate nucleolar and telomeric silencing in yeast. EMBO J.

[B9] Vergnes B, Sereno D, Madjidian-Sereno N, Lemesre JL, Ouaissi A (2002). Cytoplasmic SIR2 homologue overexpression promotes survival of *Leishmania *parasites by preventing programmed cell death. Gene.

[B10] Afshar G, Murnane JP (1999). Characterization of a human gene with sequence homology to *Saccharomyces cerevisiae *SIR2. Gene.

[B11] Gasser SM, Cockell MM (2001). The molecular biology of the SIR proteins. Gene.

[B12] Kaeberlein M, McVey M, Guarente L (1999). The SIR2/3/4 complex and SIR2 alone promote longevity in *Saccharomyces cerevisiae *by two different mechanisms. Genes Dev.

[B13] Tissenbaum HA, Guarente L (2002). Model organisms as a guide to mammalian aging. Dev Cell.

[B14] Cohen HY, Miller C, Bitterman KJ, Wall NR, Hekking B, Kessler B, Howitz KT, Gorospe M, de Cabo R, Sinclair DA (2004). Calorie restriction promotes mammalian cell survival by inducing the SIRT1 deacetylase. Science.

[B15] Giannakou ME, Partridge L (2004). The interaction between FOXO and SIRT1: tipping the balance towards survival. Trends Cell biol.

[B16] Guarente L (2000). Sir2 links chromatin silencing, metabolism, and aging. Genes Dev.

[B17] Zemzoumi K, Sereno D, Francois C, Guilvard E, Lemesre JL, Ouaissi A (1998). *Leishmania major *: cell type dependent distribution of a 43 kDa antigen related to silent information regulatory-2 protein family. Biol Cell.

[B18] Velge P, Ouaissi MA, Cornette J, Afchain D, Capron A (1988). Identification and isolation of *Trypanosoma cruzi *trypomastigote collagen-binding proteins: possible role in cell-parasite interaction. Parasitology.

[B19] Dimri GP, Lee X, Basile G, Acosta M, Scott G, Roskelley C, Medrano EE, Linskens M, Rubelj I, Pereira-Smith O, Peacocke M, Campisi J (1995). A biomarker that identifies senescent human cells in culture and in aging skin *in vivo*. Proc Natl Acad Sci U S A.

[B20] Ouaissi A (2003). Apoptosis-like death in trypanosomatids: search for putative pathways and genes involved. Kinetoplastid Biol Dis.

[B21] Yang W, Boss WF (1994). Regulation of phosphatidylinositol 4-kinase by the protein activator PIK-149. J Biol Chem.

[B22] Hayflick L (1965). The limited *in vitro *lifetime of human diploid cell strains. Exp Cell Res.

[B23] Bogdan C, Donhauser N, Doring R, Rollinghoff M, Diefenbach A, Rittig MG (2000). Fibroblasts as host cells in latent leishmaniosis. J Exp Med.

[B24] Chang KP (1978). Leishmania infection of human skin fibroblasts *in vitro *: absence of phagolysosomal fusion after induced phagocytosis of promastigotes, and their intracellular transformation. Am J Trop Med Hyg.

[B25] Dedet JP, Ryter A, Vogt E, Hosli P, da Silva LP (1983). Uptake and killing of *Leishmania mexicana amazonensis *amastigotes by human skin fibroblasts. Ann Trop Med Parasitol.

[B26] Marcotte R, Lacelle C, Wang E (2004). Senescent fibroblasts resist apoptosis by down regulating caspase-3. Mech Ageing Dev.

[B27] Nandan D, Yi T, Lopez M, Lai C, Reiner NE (2002). *Leishmania *EF-1alpha activates the Src homology 2 domain containing tyrosine phosphatase SHP-1 leading to macrophage deactivation. J Biol Chem.

[B28] Rittig MG, Bogdan C (2000). Leishmania-host-cell interaction: complexities and alternative views. Parasitol Today.

